# The Removal of Residual Concentration of Hazardous Metals in Wastewater from a Neutralization Station Using Biosorbent—A Case Study Company Gutra, Czech Republic

**DOI:** 10.3390/ijerph17197225

**Published:** 2020-10-02

**Authors:** Eva Pertile, Vojtech Vaclavik, Tomas Dvorsky, Silvie Heviankova

**Affiliations:** Faculty of Mining and Geology, Department of Environmental Engineering, VSB—Technical University of Ostrava, 708 00 Ostrava, Czech Republic; eva.pertile@vsb.cz (E.P.); tomas.dvorsky@vsb.cz (T.D.); silvie.heviankova@vsb.cz (S.H.)

**Keywords:** biosorption, desorption, hazardous waste, neutralization station, nickel, zinc, copper, iron

## Abstract

This article deals with the possibility of using a biosorbent in the form of a mixture of cones from coniferous trees to remove the residual concentration of hazardous metals contained in hazardous waste, which is disposed of in a neutralization station. The efficiency of the tested biosorbent in removing Ni, Zn, Cu, and Fe was monitored here. Laboratory research was carried out before the actual testing of the biosorbent directly in the operation of the neutralization station. With regard to the planned use of the biosorbent in the operational test, the laboratory experiments were performed in a batch mode and for the most problematic metals (Ni and Zn). The laboratory tests with real wastewater have shown that the biosorbent can be used to remove hazardous metals. Under the given conditions, 96% of Ni and 19% of Zn were removed after 20 min when using NaOH activated biosorbent with the concentration of 0.1 mol L^−1^. The inactivated biosorbent removed 93% of Ni and 31% of Zn. The tested biosorbent was also successful during the operational tests. The inactivated biosorbent was applied due to the financial costs. It was used for the pre-treatment of hazardous waste in a preparation tank, where a significant reduction in the concentration of hazardous metals occurred, but the values of Ni, Cu, and Zn still failed to meet the emission limits. After 72 h, we measured 10 mg L^−1^ from the original 4,056 mg L^−1^ of Ni, 1 mg L^−1^ from the original 2,252 mg L^−1^ of Cu, 1 mg L^−1^ from the original 4,020 mg L^–1^ of Zn, and 7 mg L^−1^ from the original 1,853 mg L^−1^ of Fe. However, even after neutralization, the treated water did not meet the emission limits for discharging into the sewer system. The biosorbent was, therefore, used in the filtration unit as well, which was placed in front of the Parshall flume. After passing through the filtration unit, the concentrations of all the monitored parameters were reduced to a minimum, and the values met the prescribed emission limits. The biosorbent was further used to thicken the residual sludge in the waste pre-treatment tank, which contributed to a significant reduction in the overall cost of disposing of residual hazardous waste. This waste was converted from liquid to solid-state.

## 1. Introduction

Water plays an important role in the world economy. However, this natural resource is becoming scarce in many places, and its unavailability is a major social and economic problem. That is why it has recently been more and more important to protect freshwater bodies for a healthy population. In practice, industrialization is responsible for environmental pollution to a greater extent, especially in the case of water in lakes and rivers, which are congested with large amounts of toxic substances. Because these metals are not biodegradable, they tend to accumulate in the environment [1].

Conventional methods used to remove metal ions, such as chemical precipitation or membrane filtration, are extremely expensive when large amounts of contaminated water are treated and are often inefficient with low metal concentrations. They also produce large amounts of sludge and other toxic products that require subsequent disposal. The disadvantages also include high agent and energy requirements and aggregation of metal precipitates, as well as membrane contamination [2,3,4,5].

Biosorption (and bioaccumulation) seem to be their suitable ecological alternatives that can be used to remove hazardous metal ions from industrial wastewater. Biosorption is a reversible, relatively rapid process that involves the binding of ions present in water solution to characteristic groups located on the surface of the biosorbent [6]. Several natural materials have already been proposed for potential biosorbents that can be used to remove hazardous metals from the water environment. They include inanimate microbial biomass, plant material, agricultural waste, and industrial by-products, biopolymers, etc. [7,8,9,10,11,12]. Some industrial by-products may also be used for wastewater treatment. For example, the food industry disposes of large amounts of waste and by-products. Unfortunately, the costs of their disposal are sometimes high. The use of these industrial waste materials as efficient biosorbents (practically with minimal cost) for wastewater treatment can thus solve two problems at the same time—waste disposal and wastewater treatment [13]. There has also been a great deal of interest in the possibility of removing pollutants from wastewater using agricultural waste/by-products as potential biosorbents. Agricultural waste, especially wastes with a high percentage of cellulose and lignin, contains polar functional groups such as amino groups, carbonyl, alcohol, phenol, and ether groups, which have a high binding potential to metal [14,15,16,17,18,19]. These groups provide a free electron pair and, therefore, form complexes with metal ions present in the solution [20]. Due to their unique chemical composition (presence of hemicellulose, lipids, lignin, monosaccharides, and starch with different characteristic groups) as well as their availability, the use of agricultural waste seems to be a viable option for heavy metal removal. The advantages of biosorption include simple operation, no additional nutrient requirements, low sludge formation, low operating costs, high efficiency, possible biosorbent regeneration, and no increase in chemical oxygen demand (COD) of water, which otherwise represent a major limitation for most conventional techniques [21]. Biosorption can also be used to remove contaminants in diluted concentrations. It is, therefore, particularly important when we deal with the removal of heavy metals due to their toxicity, which is at the level of µg L^−1^ [6]. Table 1 presents selected pilot studies focused on the removal of the metals of interest from aqueous solutions using the biosorption method.

It should be noted that there are other environmentally friendly methods for removing heavy metals from aqueous solutions, such as electrocoagulation, which is suitable for the treatment of river water or the removal of iron from drinking water. In this technique, pollutant removal is done without adding chemicals, which is why it greatly reduces the sludge produced and consequently reduces the cost of sludge handling [38,39].

The research objective was to verify the possibility of using a mixture of cones as potential biosorbent to remove the residual concentrations of Ni, Zn, Cu, and Fe in the treated industrial wastewater from the electroplating plant in such a way to meet the required emission limits for water discharged into the sewer system of the company.

## 2. Materials and Methods 

### 2.1. The Method Used for Wastewater Treatment in the Company

Operational tests were performed in a company that specializes in the treatment and removal of toxic metals contained in various types of hazardous and other waste materials. The waste first undergoes the physical-chemical treatment, during which sedimentary sludge and wastewater were separated. The wastewater composition must meet the conditions for entry into the industrial wastewater treatment plant, which was situated on the site.

Hazardous wastes and other wastes from the suppliers were discharged into the reaction tanks directly from the tank truck. The retention time here was approximately 2 h. One tank was being filled, while neutralization took place in the other one. Compressed air was fed to the bottom of both tanks to mix their contents. The actual neutralization was carried out by adding Ca(OH)_2_ or NaOH. The goal was to achieve the final pH value within the range of 7.5 to 8.5 to make the neutralization itself effective. Regular pH checks were, therefore, very important. After the neutralization was completed, the neutralized water was transferred to the sedimentation tank using a pump. Its function was based on the principle that the newly pumped content of neutralized water pushes out the same volume of already treated water into the company sewer system. The residual content of hazardous metals must always be checked before the discharge. Whether the wastewater does not exceed the given emission limit of the monitored hydrochemical indicator, which was prescribed for technological neutralization equipment, was evaluated according to Government Decree No. 401/2015 Coll. on indicators and values of permissible pollution of surface waters and wastewaters, requirements for permits for the discharge of wastewaters into surface waters and sewers and on sensitive areas. For these hydrochemical indicators, the emission standards of supplied and discharged wastewater were set by the Regional Office of the Department of the Environment and North Bohemian Waterworks and Sewerage, which were specified in the Operating Rules of company GUTRA.

The real samples of industrial wastewater, which were tested in the research, came from the treatment of hazardous waste with the catalog number 11 01 05 (acid pickling solutions) from the company REPON s.r.o. Žatec, Czech Republic, which deals with surface treatment of metals. This was wastewater from a galvanizing process, which was not specified. The company GUTRA as the recipient of the waste, deals only with the content of hazardous metals (Cu, Ni, Zn, and Fe), pH, chemical oxygen demand (COD), dissolved inorganic substances (DIS), and insoluble substances (IS).

### 2.2. Monitored Parameters and the Methodology 

It was necessary to monitor and adjust the pH value during the experiment because it plays a significant role during the adsorption of hazardous metals on the surface of the biosorbent used. The measurement was performed using a pH-meter WTW 3110 (Xylem Analytics Germany Sales GmbH & Co. KG, WTW, Weilheim, Germany).

The concentration of hazardous metals was determined spectrophotometrically using a HACH DR 2800 spectrophotometer (HACH LANGE GmbH, Berlin, Germany). The samples had to be diluted for measurement to suit the scope of the method used. Each metal was determined separately, and the measurements were repeated a total of three times, thus that the values given represented the average of the values from the 3 measurements. The measurement error did not exceed 3%. These methods followed the HACH Water Analysis Handbook [40]. The concentration of Ni (II) was determined using the HACH LCK (HACH LANGE GmbH, Berlin, Germany) 537 method within the range of 0.05 to 1.00 mg L^−1^ at the wavelength of 463 nm in glass cells with an optical path of 2 cm. In the presence of an oxidizing agent, nickel ions reacted with dimethylglyoxime in an alkaline solution to form an orange-brown-colored complex [40]. The concentration of Zn (II) was determined using HACH LCK 360 method within the range of 0.2 to 6 mg L^−1^ at a wavelength of 490 nm in glass cells with an optical path of 2 cm. Zinc ions form a water-soluble orange-red complex with 4-(2-pyridylazo)-resorcin (PAR) at pH 6−11 [40]. Fe_tot_ (total iron) concentration was determined using HACH 8008 method within the range of 0.02 to 3 mg L^−1^ at a wavelength of 510 nm in glass cells with an optical path of 2 cm. FerroVer Iron Reagent (HACH LANGE GmbH, Berlin, Germany) reacts with all soluble iron and most insoluble forms of iron in the sample to produce soluble ferrous iron. This reacts with 1,10-phenanthroline indicator in the reagent to form an orange color in proportion to the iron concentration [40]. The concentration of Cu (II) was determined using HACH 8506 method (CuVer 1) within the range of 0.04 to 5 mg L^−1^ at a wavelength of 560 nm in glass cells with an optical path of 2 cm. Copper in the sample reacts with the salt of bicinchoninic acid contained in CuVer 1 to form a purple-colored complex in proportion to the copper concentration [40].

The measured values of both input and output concentrations within the frame of the laboratory model experiments were used to calculate the adsorption capacity *q* using Equation (1) [41]:

(1) $$q_{e}=\frac{V\left(c_{i}-c_{f}\right)}{m}$$

where: *q*: metal adsorption in mg g^−1^; *V*: volume of the model metal solution, in L; *c_i_*: input concentration of the model metal solution in mg L^−1^; *c_f_*: output concentration of the model metal solution in mg L^−1^; *m*: amount of activated biosorbent added to the model metal solution in g.

The biosorption efficiency (*R* in %) for the given metal was calculated based on Equation (2) [41], the parameters have the same meaning as in Equation (1):

(2) $$R\;\%=\frac{c_{i}-c_{f}}{c_{i}}\times 100$$

### 2.3. Selected Biosorbent

Simple criteria were chosen for the selection of the biosorbent. The main factor for the selection was that it had to be easily available and that its preparation had to require the lowest possible cost. Cones of coniferous trees, which were easily available, met this criterion, and their good sorption capacity for the main hazardous metals (Ni and Zn) was demonstrated in laboratory experiments. Their lavish occurrence in the Czech Republic was related to the abundant source of unused cone biomass as a renewable resource. The biomass of conifer fruits was essentially forest waste in itself, and thus readily available potential biosorbent. Although they were used in small quantities for decorative purposes, for feeding horses or they were crushed and added to mulch, in most cases, the cones remained in the forests. The ripe cone was made up of epidermis and sclerenchymatous cells, which contained cellulose, hemicellulose, and lignin in their cell walls. There were also natural resins and tannins. From the chemical point of view, tannins were large polyphenolic compounds that contained hydroxyl and carboxyl groups. That was why the cone biomass can provide the specified binding groups to the metal–biosorbent bond.

The treatment of the cone mixture consisted of mechanical and physical ones. The collected cones were first cleaned of any coarse impurities and then dried freely in the air to make their further mechanical treatment easier. For laboratory experiments, which were carried out in the laboratory of the Department of Environmental Engineering of VŠB—Technical University of Ostrava, the mechanically roughly treated biosorbent was further crushed to a smaller grain fraction (fraction 2/5 mm). A Raptor laboratory electric grinder was used for this purpose. The ground samples of the conifer cone mixture were then sieved to the required grain size fraction on the Retch company stainless steel sieves. The grain size fraction of 2/5 mm was selected (maintaining the criterion of the lowest possible sorbent treatment costs) based on the reference resources and the methodology applied to Department of Environmental Engineering for the study of the biosorption mechanism as well as the use of the biosorbent in operation. To make the adsorption more effective, chemical modification of adsorbents may ensure the accessibility of the functional groups on the surface of the adsorbent. Sodium hydroxide was selected as the activating agent at the concentration of 0.1 mol L^–1^. For each gram of the adsorbent, 20 mL of an activating solution were used. We mixed the samples using an IKAKS 4000i (IKA-Werke GmbH & Co. KG, Staufen, Germany) control laboratory shaker for 30 min at 150 rpm. After activation, the adsorbents were filtered and subsequently washed with distilled water several times to remove the residual activating agent. The final pH was measured using ION340i pH meter (Xylem Analytics Germany Sales GmbH & Co. KG, WTW, Weilheim, Germany). Subsequently, the activated adsorbent was dried in ECOCELL ECO oven (MMM Medcenter Einrichtungen GmbH, München, Germany) at 65 ± 1 °C to constant weight to guarantee its exact weight to determine the equilibrium adsorption process.

The cone mixture was crushed into smaller particles for the operational tests (0/10 mm fraction) using ALKO H2200 mobile chipper (AL-KO KOBER SE, Kötz, Germany). Chemical activation of the biosorbent was not performed in this experiment, due to the additional possible loading of the biosorbent and also the economic costs of its preparation.

### 2.4. Methodology and Experiment Conditions in Batch Mode

Laboratory studies had been carried out in a batch mode before the experiments were started directly in the operation of the neutralization station. The conditions of the experiment, which were based on the experience of studying biosorption at Department of Environmental Engineering VŠB—Technical University of Ostrava, are presented in the following Table 2.

## 3. Results and Discussion

The laboratory experiments were carried out in a batch mode with regard to the planned use of the biosorbent in the operational test, i.e., for the pre-treatment of hazardous waste and its subsequent final purification after neutralization. That is why the existing results and experience gained from the study of the biosorption mechanism in the so-called batch mode for a cone mixture and the individual metals were used. Because the treated waste was hazardous (acid pickling solutions), special attention was paid to the effectiveness of the applied biosorbent for the following hazardous metals: Ni; Zn; Cu, and Fe_tot_. Since these metals were studied only individually, not together, the most suitable experimental conditions from these studies were used. They included pH, exposure time, temperature, chemical activation of the biosorbent used, and grain size adjustment.

### 3.1. Results of Laboratory Experiments

The used biosorbent has already been tested for a wide range of metals at Department of Environmental Engineering VŠB—Technical University of Ostrava. The concentration of the biosorbent was 20 g L^−1^, the initial concentration of metal was 100 mg L^−1^, and it was operated at room temperature. The stirring speed of 150 rpm was used in all the experiments. The chosen mixing speed thus provided the best homogeneity of the mixture suspension. At higher speeds, there was a risk that the suspension might not be homogeneous, and the biosorption of metals could thus be adversely affected. Modeling of adsorption kinetics was applied to determine the most ideal contact time that was necessary for the biosorption of metals and equilibration between the two phases of biosorbent–adsorbate. 

Based on the study of kinetics, the optimal size of the biosorbent and the method of its chemical activation (strengthening of negatively charged functional groups on the surface of the biosorbent) were chosen as well. Thus, the optimal conditions were experimentally determined only for the chemically modified biosorbent that showed the highest efficiency of the removal of metals from the model solution during the modeling of the kinetics of biosorption. The effect of pH on the process of biosorption was studied with respect to the nature of the studied metals (tested for the pH range of 4.0 to 7.0; with higher pH, metals already begin to precipitate due to the formation of MeOH). The individual values of pH of the model solutions of metals were maintained using buffers, which were used for the preparation of the model solution of metals. Two models were used to describe the isotherms: Langmuier and Freundlich. The results indicated that the biosorption process was relatively fast (20 min), and the equilibrium was reached after about 40–50 min of contact. The kinetics and equilibrium data were best-fitted pseudo-second-order and Langmuir isotherm indication monolayer chemisorption with the maximum capacity *q*_max_ (The maximum adsorption amount per unit of the weight of the sorbent, which forms a complete monolayer on the surface) see in Table 3.

However, the tests were carried out only for the individual hazardous metals in the model solutions, not for a real sample. This is because other ions present in the solution can adversely affect the biosorption process by competing for the binding spots of the used biosorbent. If more metals are present, lower sorption of each of them can be assumed. However, in reality, the sorption capacity of multiple metals is often comparable to the sorption of single-type solutions. This is most likely related to the existence of different binding groups with different affinities for the given metal ions, which do not interact with each other. Wastewater after the neutralization of hazardous waste was, therefore, first tested within the scope of the laboratory experiments, namely, to detect the content of the priority hazardous metals, i.e., Ni and Zn. The content of the monitored metals after the neutralization of the processed hazardous waste is presented in Table 4.

The pH of the treated wastewater (after the neutralization process) was 7.0. Based on the experience gained during the study of biosorption of the studied biosorbent (a mixture of cones) in model solutions, the ideal pH value for the most efficient sorption of both hazardous metals is 6.0. That is why the pH value of the neutralized wastewater was adjusted to this value using HCl with a concentration of 2.0 mol L^−1^ before the actual experiment. The pH value was monitored during the experiment, which is why it can be stated that there was no change in the pH. Regarding the sorbed metals, NaOH-activated biosorbent with the concentration of 0.1 mol L^−^^1^ was used for the experiment to strengthen the negatively charged characteristic groups on the surface of the biosorbent. This made the sorption of cations of the metals in question easier. The condition of the lowest possible economic cost of the operation and the increase of contamination by adding other chemicals meant that the effectiveness of the biosorbent without chemical treatment was tested as well. A grain size fraction of 2/5 mm was tested with regard to the target application of the biosorbent. It was, therefore, not necessary to further process the cone mixture by sieving it into a smaller grain size fraction. This larger grain fraction was also beneficial because in the final stage of real wastewater treatment after neutralization, the cone mixture will be used for the final purification of the already treated water before its discharge to the sewer system, thus that the hazardous metals meet the prescribed emission limit. The storage of the sorbent in gunny-sacks seems to be the most suitable and, at the same time, the easiest method for further handling of the sorbent. If biosorbent with small grain fractions had been used, it could have leaked from the bags into the water drain and the sewer system.

The methodology used for the actual laboratory experiment was verified at Department of Environmental Engineering VŠB—Technical University of Ostrava. Under the given conditions, a total of 96% (q_20_ = 34.3 mg g^−1^) Ni and 19% (q_20_ = 0.04 mg g^−1^) Zn were removed using the NaOH activated biosorbent with the concentration of 0.1 mol L^−1^ after 20 min. When inactivated biosorbent was used, a total of 93% (q_20_ = 33.5 mg g^−1^) Ni and 31% (q_20_ = 0.06 mg g^−1^) Zn were removed after 20 min under the given conditions. By comparing the sorption capacity of the chemically activated biosorbent (q_20_ = 0.004 mg g^−1^) and the non-activated biosorbent (q_20_ = 0.006 mg g^−1^) and concerning the determination error, it can be stated that there were no significant changes.

### 3.2. Outcomes of the Operational Experiment 

The laboratory experiments concerning the effectiveness of the studied biosorbent to reduce the concentration of the riskiest metals in hazardous waste (Ni and Zn) were followed by operational tests. Biosorbent that was not chemically activated was chosen for the operational experiment, with regard to the lowest possible load of the process of reducing the concentration of the monitored metals by further chemical pollution. Other hazardous metals, which are contained in the treated waste, were also monitored due to the composition of the hazardous waste. They were Cu and Fe. Other hydrochemical parameters, such as dissolved inorganic substances (DIS) and insoluble substances (IS), were also monitored during the operational test. In the case of the treated water discharged after the final filtration through biosorbent, the chemical oxygen demand (COD) was also monitored.

The biggest problem as far as the treatment of this type of hazardous waste, consisting of very strong acids with a high content of toxic metals, is concerned was the fact that it could not be processed efficiently in the neutralization station to achieve the separation of hazardous metals from water. In general, this hazardous waste is very difficult to process, and its treatment is relatively very expensive and also very time-consuming as a result of that. The price for the processing of this type of waste can thus rise well above the price the customer pays for its disposal.

With respect to the problem described above, the studied biosorbent was also used for the pre-treatment of this type of waste as part of the subsequent strengthening of the neutralization process. This is the reason why the crushed biosorbent was also poured into the preparation tank. In order to achieve an ideal contact of the biosorbent with the disposed waste and the neutralizing agent, the tank was mixed using an excavator. For practical reasons, the neutralization tanks cannot be cleaned of residual sludge after each waste dose. An analysis of the contents of the tank was also performed before adding the disposed waste as a result of this fact. The operational testing of the biosorbent included the monitoring of its effectiveness after 24 h, 48 h, and 72 h. After 24 h, the content of the reaction tank was always mixed using an excavator to ensure sufficient contact of the biosorbent with the treated hazardous waste. The resulting values of the individual analyzes, including the emission limits, are presented in Table 5.

The results of the operational experiment show that the use of biosorbent for the pre-treatment of hazardous waste was effective. There was a significant reduction in all the monitored parameters, but despite that, the required emission limits were not reached. However, there was a realistic assumption that these lower concentrations would be much easier to remove within the process of further treatment in sedimentation tanks. The added value of this pre-treatment of hazardous waste can be the fact that the use of the biosorbent in the neutralization process thickens the residual sludge after neutralization, thus reducing the cost of further disposal. The company does not need to dispose of the generated waste after neutralization in the form of liquid waste, but it can dispose of it in the form of a solid waste, which is much more economically beneficial. The company pays approximately 214 € for the disposal of 1 ton of liquid hazardous waste. On the contrary, when disposing of solid hazardous waste, the company pays only approximately €25/1 t.

**Figure 1:**
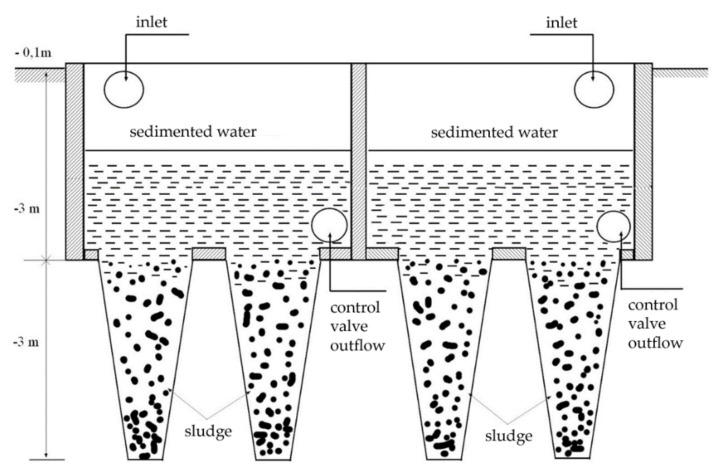
Scheme of the designed sedimentation tank and its mechanism.

After the physical–chemical treatment, during which the sedimented sludge and also the wastewater were separated, the neutralized water was transferred to the sedimentation tank using the FEKO-SIEMENS (Siemens Aktiengesellschaft, Munich, Germany) pump (Figure 1). The treated wastewater remained here for 48 h. After this time, a laboratory analysis must be performed in order to check the permissible emission limits for the discharge of the treated wastewater into the company sewer system.

A laboratory inspection revealed that the treated wastewater still did not meet the permitted emission limit for its discharge into the company sewer system (see Table 6).

Based on the fact described above, the treated water passed through a filtration unit with the biosorbent again, where only the residual concentration of the monitored hazardous metals was to be captured. Approximately 1 t of Ca(OH)_2_ and 1,250 kg of cone mixture had to be added to the preparation tank to 900 L of treated hazardous waste and 8 t of sludge, which remained in the tank after the previous treatment of other waste materials. It seems to be most suitable for hazardous waste to be in contact with the biosorbent for at least 72 h in the preparation tank. The pre-treated water was then sucked off using a tank truck and taken to the neutralization station, where the actual neutralization process took place with the adjustment of the pH value of Ca(OH)_2_ to the target value of 6.5. After the completion of the neutralization process, the wastewater was transferred to sedimentation tanks. The sedimentation process itself took about 48 h. The separated treated wastewater takes advantage of gravity to flow into the sewer system.

The resulting values obtained after the final filtration treatment of the wastewater that were measured in the treated wastewater samples taken behind the Parshall flume are presented in Table 7.

The values presented in the table clearly show that the use of the studied biosorbent achieved the maximum possible efficiency of the biosorbent when the residual concentration of hazardous metals was reduced to values that fully meet the emission limits for their discharge into the industrial sewer system. In total, two bags of 50 kg of biosorbent were used, and they were placed in front of the Parshall flume.

### 3.3. Treatment and Further Utilization of the Used Biosorbent

Another possibility of using the already spent biosorbent is its reactivation using the opposite process to biosorption, which is the desorption process. The desorption conditions were tested in laboratory experiments. The spent biosorbent that had been used for the analysis was first dried to a constant weight at 60 °C to create suitable conditions for accurate weighing of the biosorbent for further treatment. The drying was carried out in a Memmert UNB 200 oven (Memmert GmbH + Co.KG, Büchenbach, Germany). This heat-treated used biosorbent was then placed in a glass column for further treatment. A total of 44.5 g of spent biosorbent was used to regenerate the biosorbent in laboratory conditions. It was produced by drying 50 g of used biosorbent. The biosorbent was first washed in a column with a regenerating agent, which was hydrochloric acid in the ratio of 1:1, for 60 min. A total of 50 mL of regeneration solution was used to regenerate it to the required amount of used biosorbent. This amount after regeneration was disposed of, and then the biosorbent in the column was washed with 100 mL of distilled water in 10 cycles.

A total of 10 samples were prepared within the scope of the biosorbent regeneration. They were subsequently subjected to an analysis to reveal the content of the residual concentration of hazardous metals. For practical reasons, only selected samples were used to analyze the content of the monitored parameters after the regeneration of the used biosorbent. These were samples no. 1 (after washing with 100 mL of distilled water); sample no. 5 (after washing with 500 mL of distilled water); sample no. 7 (after washing with 700 mL of distilled water); sample no. 9 (after washing with 900 mL of distilled water) and sample no. 10 (after washing with 1,000 mL of distilled water). The individual results are shown in Table 8.

The results presented above clearly show that the used biosorbent after regeneration with hydrochloric acid and subsequent washing of the biosorbent with distilled water in 10 cycles achieved practically 100% regeneration. This means that the used biosorbent could participate in the next biosorption process.

When the economic side of the application of the tested biosorbent is taken into consideration, it is also important to deal with its disposal or its further use. If the company decides to regenerate the used biosorbent, then it is necessary to include the costs of rinsing water, acid, disposal of wastewater after regeneration, energy, and work when calculating the regeneration costs. The cost of biosorbent regeneration would then amount to approximately €1,130, which is not very interesting economically. Even if the company disposed of the used biosorbent without further use, i.e., as solid waste, the costs would be more advantageous for the company than its regeneration. As discussed above, the spent biosorbent can be used to thicken the liquid waste that is generated during the pre-treatment of hazardous waste before the actual neutralization process. The company can thus achieve a very interesting saving as a result of the much lower price for the disposal of solid waste (approximately €25/1 t) compared to the disposal of liquid waste (approximately €214/1 t).

## 4. Conclusions

Both the laboratory experiments and the operational tests have shown that the mixture of cones from coniferous trees is suitable for removing hazardous metals from wastewater. This biosorbent has proved very successful, both for the pre-treatment of hazardous waste and for the final purification of the residual concentration of metals before discharging water into the sewer system.

Based on the operational test, it can be stated that the mixture of cones from coniferous trees is usable for the final purification of the total of 10,000 L of wastewater thus that it meets the emission limits for discharging into the sewer system. After that, the effectiveness of the biosorbent decreases and it is necessary to replace it.

Practical experience with hazardous waste treatment made it possible to design technology for the use of low-cost biosorbent (a mixture of cones from coniferous trees) to remove hazardous metals from disposed of hazardous waste, without compromising the emission limits when discharging wastewater to the sewer system and subsequently to a wastewater treatment plant.

Biosorption can be integrated with conventional water treatment. This is especially important in the case of pollutants, such as heavy metals, whose effects are visible even at the ppb (parts per billion) level. Compared to conventional technologies, it can be stated that biosorption is beneficial for the treatment of contaminated water. Its potential has been proven both at the laboratory and pilot levels and the actual outflow/discharge of polluted wastewater. Another advantage of the designed technology is that the described biosorbent can also be used to thicken the residual sludge in the waste pre-treatment tank, which contributes to reducing the overall cost of removing residual hazardous waste. This waste is converted from a liquid to a solid one. The company can, therefore, dispose of the solid waste generated after the neutralization, which is much more beneficial from an economic point of view.

## Figures and Tables

**Table 1:** Selected pilot studies focused on the removal of the metals of interest from aqueous solutions using the biosorption method.

Biosorbent	Metal	Adsorption Capacity/Efficiency mg g^−1^/%	pH	References
Sugar cane waste	Ni	2 mg g^−1^	5	[22]
Grapefruit peel	Ni	46 mg g^−1^	5	[23]
Peanut shells—chemical modification (HNO_3_, NaOH)	Ni	0.17 mg g^−1^; 57%	7	[24]
Bark from *Eriobotrya japonica*—chemical modification (0.1 M NaOH)	Ni	28 mg g^−1^	6	[25]
Pistachio shells	Ni	14 mg g^−1^, 75%	4–6	[26]
Tapioca peels	Ni	20 mg g^−1^, 71%	5	[27]
*Lythrum salicaria* L.	Ni	9 mg g^−1^	7	[28]
Papaya wood	Cu, Zn	95%, 66.8%	5	[29]
Wheat husks	Cu	99%	5–6	[30]
Leaves of *Nyctanthes arbor-tristis* powder	Ni, Cu	55 mg g^−1^, 44 mg g^−1^,	6 5	[31]
Oak sawdust	Cu Ni	93% 82%	4 8	[32]
Walnut shells	Ni; Zn	4 mg g^−1^; 4 mg g^−1^	5	[33]
Tobacco dust	Zn	25.1 mg g^−1^	7	[34]
*Phyllanthus debilis*	Zn Ni	8.97 mg g^−1^ 11.4 mg g^−1^	5	[35]
*Ceratophyllum demersum*	Cu Zn	6.2 mg g^−1^ 14 mg g^−1^	5–6	[36]
Cine biomass of *Thuja orientalis*	Cu	19.2 mg g^−1^	5–6	[37]

**Table 2:** Conditions used for laboratory testing of cone mixture for real wastewater after neutralization.

Parameters	Value
Adjusted pH value of wastewater	6.0
Biosorbent weight	1.0000 g
Wastewater sample volume	50.0 mL
Contact time (adsorbent-adsorbate) for the batch system	20 min
Temperature	21 °C
Mixing speed during sorption	150 rpm
Grain size adjustment	2/5 mm
Chemical activation of a cone mixture	NaOH concentration 0.1 mol L^−1^ for 30 min;
	non activated

**Table 3:** Results obtained in laboratory tests.

Metal	Chemical Activation/Activation Time	Particle Size mm	*q*_20_ mg g^−1^	Sorption Efficiency %	pH	Isotherms Models	Maximum Adsorption Capacities q_max_ mg g^−1^
Ni	none	0.5–1.0	4.36	87	*	*	*
	0.1M NaOH/30 min		4.96	99	6.0	Langmuir	10.76
Zn	none	0.5–1.0	4.32	86	*	*	*
	0.1M NaOH/30 min		4.86	97	6.0	Langmuir	12.46
Cu	none	0.5–1.0	4.17	83	*	*	*
	0.1M NaOH/30 min		4.40	88	5.0	Langmuir	6.52
Fe	none	0.5–1.0	4.08	82	*	*	*
	0.1M NaOH/30 min		4.90	98	6.0	Langmuir	10.65

* not specified; q_20_ = adsorption capacity—contact time 20 min.

**Table 4:** Content of monitored hazardous metals from wastewater after neutralization.

Toxic Metal	Concentration mg L^−1^	Emission Limit [42,43] mg L^−1^
Nickel	718	0.1
Zinc	3.6	0.5

**Table 5:** Results of the analysis depending on the duration of action of the biosorbent used.

Parameters	Unit	Added Waste	Residual Sludge in the Tank	Concentration after 24 h	Concentration after 48 h	Concentration after 72 h	Limit [42,43]
pH	–	<1.0	5.5	6.1	5.9	6.0	6–9
DIS	mg L^−1^	23,856	17,465	4356	2148	1745	1200
IS	mg L^−1^	3298	2417	235	188	122	350
Hazardous metals							
Ni	mg L^−1^	4056	253	24	18	10	0.1
Fe_tot_	mg L^−1^	1853	55	28	14	7	10
Cu	mg L^−1^	2252	198	13	3	1	0.1
Zn	mg L^−1^	4020	421	18	3	1	0.5

DIS—dissolved inorganic substances; IS—insoluble substances; Fe_tot_—total Iron.

**Table 6:** Results of the analysis dealing with discharge from the sedimentation tank into the sewer system.

Parameters	Unit	Value	Emission Limit [42,43]
pH	–	7.3	6–9
COD Cr	mg L^−1^	589	800
DIS	mg L^−1^	345	1,200
IS	mg L^−1^	78	350
Cu	mg L^−1^	**0.2** ^1^	0.1
Ni	mg L^−1^	**0.3**	0.1
Zn	mg L^−1^	**0.7**	0.5
Fe_tot_	mg L^−1^	1.2	10

^1^ the values exceeding the emission limits for discharge into the company sewer system are marked in bold; COD Cr - chemical oxygen demand.

**Table 7:** Values after final pre-treatment before discharging wastewater into the company sewer system.

Parameters	Units	Results	Emission Limit [42,43]
pH	–	6.5	6–9
COD	mg L^−1^	523	800
DIS	mg L^−1^	265	1,200
IS	mg L^−1^	54	350
Cu	mg L^−1^	<0.1	0.1
Ni	mg L^−1^	<0.1	0.1
Zn	mg L^−1^	0.2	0.5
Fe_tot_	mg L^−1^	0.7	10

**Table 8:** Results of the individual parameters monitored during the regeneration of spent biosorbent—a mixture of cones of coniferous trees.

Sample	Ni mg L^−1^	Zn mg L^−1^	Cu mg L^−1^
1	0.74	1.11	0.42
5	0.49	0.98	0.18
7	0.15	0.61	0.12
9	0.01	0.15	0.04
10	0	0	0
